# Influence of Contextual Variables on Physical and Technical Performance in Male Amateur Basketball: A Case Study

**DOI:** 10.3390/ijerph17041193

**Published:** 2020-02-13

**Authors:** Antonio Fernández-Leo, Carlos D. Gómez-Carmona, Javier García-Rubio, Sergio J. Ibáñez

**Affiliations:** 1 Training Optimization and Sports Performance Research Group (GOERD), Sport Science Faculty, University of Extremadura, 10005 Caceres, Spain; afernandtg@alumnos.unex.es (A.F.-L.); sibanez@unex.es (S.J.I.); 2 Facultad de Educación, Universidad Autónoma de Chile, Santiago 7500912, Chile

**Keywords:** basketball, amateurs, internal load, external load, performance indicators

## Abstract

Currently, most basketball research is focused on professional and elite players. Studies at the amateur level are important to explain the physical and technical demands of competition and thus improve players’ and teams’ performance. The purpose of the present study was to describe the competitive demands of an amateur-level basketball team and to analyze the influence of different situational variables on the physical and technical performance indicators. Eleven amateur senior basketball players participated in six official final-round games during the 2018/2019 season. External, internal load, and notational analysis were registered by inertial devices, heart rate bands, and video analysis. The Kruskal-Wallis H-test was applied for comparisons based on playing positions, periods, and final quarter game outcome, with the post hoc comparison accomplished by a Mann-Whitney U test. The Spearman correlation coefficient was realized for the relational analysis. The results showed that: (a) guards covered more volume of displacements (effective on-court time: *p* < 0.01, $E_{R}^{2}$ = 0.05; steps/min: *p* < 0.01, $E_{R}^{2}$ = 0.28) and the centers performed competitive actions of higher load ([>8G] Imp/min: *p* < 0.01, $E_{R}^{2}$ = 0.20; jumps/min: *p* < 0.01, $E_{R}^{2}$ = 0.33); (b) a performance decreasing was found between the first and second half of the game; (c) in balanced matches there was the most individual technical performance (PIR/min: *p* < 0.98, $E_{R}^{2}$ = 0.01), while in the unbalanced games more high-intensity impacts were seen ([>8G] Imp/min: *p* < 0.01, $E_{R}^{2}$ = 0.07). The situational variables analyzed had an influence on athletic performance in amateur senior basketball players and should be considered for designing training sessions and planning strategies during official matches.

## 1. Introduction

Basketball is an invasion team sport with simultaneous participation in a non-divided court that presents a great number of attack-defense transitions and technical-tactical actions [1]. These high-intensity, intermittent efforts require that players present high agility and power levels to realize intense acceleration and decelerations, intending to achieve an advantage during competition [2,3]. The monitoring and subsequent analysis of the players’ workload provide relevant information to understand the physical, technical and tactical demands of basketball players for training design that optimizes the performance [4,5]. This information should be used by technical staff to manage and design training sessions according to the specific players’ competition workload [6,7].

In recent years, thanks to technological advances, electronic performance and tracking systems (EPTS) have been developed. These devices include microtechnology (accelerometers, gyroscopes, magnetometers) and outdoor tracking systems such as global navigation satellite systems (GNSS), or indoor ones such as ultra-wideband (UWB) radiofrequency local position, for workload monitoring [5]. These devices have obtained satisfactory results in accuracy, reliability and validity assessments to characterize the basketball workload in competition and training sessions [8].

With these systems, the evaluation of internal and external workloads during training and competition has been raised as an issue [8]. The internal workload is the stress and physiological reaction facing a stimulus and can be measured by heart rate telemetry (HR), blood lactate levels and rated-perceived exertion scales (RPE) [4,9,10]. The external workload is defined as the locomotor and mechanical stress produced by the activity [11]. It is classified into kinematical and neuromuscular load, with the first related to displacements and their intensity, as registered by tracking systems [12], while the second is related to the forces performed by the players as a result of the interaction with the gravity and the teammates/opponents (impacts, jumps, etc.) registered through triaxial accelerometers [13]. According to a recent review by Stojanović et al. [3], during a basketball game, players have an intensity of over 85% HR_MAX_ (81.9%–89.1%), with elevated blood lactate levels from rapid glycolysis (2.7–6.8 mmoL); guards present the highest demands with respect to forwards and centers [14] and have higher values in the first half due to fatigue developing throughout the game [10].

Due to technological advances in inertial devices and the development of a vast quantity of variables that quantify external load (Player Load, Body Load, Impulse Load, Total Load, Total Acceleration, etc.) [15], the lack of research about the relationships, uses and validity of each variable for each sport (individual vs. team, indoor vs. outdoor, etc.) creates noise and error in the interpretation of the information. The need for professionals, such as data analysts or sport scientists, is critical to choose the most accurate variables in each context and to improve performance [16]. Still, specific measurements of each variable and the relationships between them is essential to advance knowledge in the sport field.

In this sense, performance indicators and contextual variables are all actions and game situations that could influence players’ performance during a basketball game [17]. Numerous studies indicated the influence of contextual variables and performance indicators in internal and external workload in elite-level clubs or national teams related to: (a) playing position, where guards covered higher distance and at more intensity than forwards and centers [7,18,19]; (b) the acute and chronic fatigue in quarter workload dynamics and season game dynamics [7,19]; (c) the influence of quarter-final points (balances vs. unbalanced games) [20]; and (d) the opponent level [7].

The available literature is, mostly, focused on elite-level basketball players and competitions. Few studies analyzed amateur basketball [18,21,22], and only Reina et al. [18] showed the workload demands during competition for female players. However, most of the player’s licenses belong to amateur basketball. Technical and physical levels (internal and external workload profiles) in the amateur category could be different than in the elite category, and it would not be correct to use the physical and technical profiles for elite-level basketball players. Therefore, it is necessary to categorize for the first time the physical and technical profile demands of basketball players at this level, as well as the interaction between the variables. The purposes of the present research were: (a) to describe the physical and technical profile of amateur basketball players; (b) to analyze the effect of different contextual variables such as playing positions, quarter periods, difference in points accumulated in the various quarters, and difference in points between each quarter in terms of physical and technical performance; and (c) to investigate the relationship between physical (internal and external workload) and technical performance in male amateur basketball. Thus, it is hypothesized that contextual variables will have a different level of influence on the physical and technical performance and we will not be able to identify a relationship between technical and physical performance.

## 2. Materials and Methods

### 2.1. Study Design and Sample

Our research employed a descriptive strategy of notational analysis to gather statistics on each game and then an associative strategy via a comparative longitudinal study using attributive variables to identify differences between groups. In this study, no intervention was performed; rather, the competition was allowed to proceed naturally [23]. The last six games played by one team in a regular season (27 January to 10 March 2019) were analyzed. All opponent teams obtained better ranking at the end of the regular season. Eight teams participated in the regular season, which had a duration of six months (October 2018 to March 2019). In the regular season, each team played against all others at home and away (14 matches). At the end of the regular season, the best four teams played the final four matches. A total of 179 sample units were analyzed, which corresponded to the participation of each player in each quarter (e.g., 1st quarter, *n* = 7, three participants played the whole match and four players played 50% of the time).

### 2.2. Participants

Eleven male amateur-level basketball players (height: 1.82 ± 0.08 cm; weight: 82.70 ± 11.99 kg; age: 18.82 ± 1.65 years) belonging to one senior team that played in a regional-level basketball club were registered. The inclusion criteria were participation in over 85% of analyzed matches and playing as line-up or substitute players in each quarter more than 50% of the time. The team staff and tournament managers gave their consent for participation in this research. Before the tournament started, all anthropometrical characteristics of the players were registered, and they signed a written consent form in accordance with the Bioethics Commission of the University (Reg. Code 67/2017). The study was conducted according to the Declaration of Helsinki (2013) guidelines.

### 2.3. Equipment

Twelve inertial devices WIMU™ (RealTrack Systems, Almería, Spain) composed of different sensors (accelerometer, magnetometer, gyroscope, etc.) were used to monitor the external workload. The accelerometers register at a 100 Hz sample frequency, and their reliability and validity were assessed previously for the vectorial sum of three-axis acceleration (*x*, *y*, *z*) [12] and Player Load [14]. The internal load was registered by HR band Garmin® (Garmin, Olathe, KS, USA). The heart rate telemetry was registered at a 4 Hz sample frequency and sent through Ant+ technology to the WIMU™ inertial device [24], where it was stored with the microsensors raw data in the 8 GB internal memory.

The time selection of quarter periods, on-court playing time and the movement playing time of each basketball player was recorded through a laptop with the software SVIVO™ (RealTrack Systems, Almería, Spain). Moreover, the video recording for the post hoc analysis of the technical individual actions of each player was performed by a Sony HDR-XR106 video camera (Sony, Minato, Tokyo, Japan). Finally, the time selection, the internal and external workload, and the video recording were imported into the software SPRO™ (RealTrack Systems, Almería, Spain) for subsequent analysis and a final data report.

### 2.4. Variables

#### 2.4.1. Independent Variables

- Game period: Data from each match were divided into four periods according to the official basketball rules: quarter 1 (Q1, *n* = 45), quarter 2 (Q2, *n* = 46), quarter 3 (Q3, *n* = 43), and quarter 4 (Q4, *n* = 45).
- Accumulated point differences of each period. Related to the accumulated difference of points at the end of each quarter, three groups were created by a k-means clustering algorithm that considered all results in the six matches registered following previous research in basketball [17,25,26,27]: (a) score differences equal to or below nine points (close games, *n* = 77), (b) score differences between 15 and 29 points (balanced games, *n* = 77) and score differences above 30 points (unbalanced games, *n* = 25).
- Final score difference of each period. The acute difference of points each quarter. Three groups were created following the procedure described at accumulated point differences of each period: (a) score difference equal to or below seven points (close games, *n* = 36), (b) score differences between eight8 and 15 points (balanced games, *n* = 57) and score differences above 15 points (unbalanced games, *n* = 76).
- Playing positions: Each basketball player has specific physical and physiological characteristics [28]. In order to explore differences by playing positions, the total sample was grouped into three basketball roles assigned by the coaches: guards (*n* = 60), forwards (*n* = 52), and centers (*n* = 67).

#### 2.4.2. Dependent Variables

- Effective on-court time. A new variable was created to analyze the effective time that the player is in motion respect to the total time on the court. This variable was calculated by the following equation:
  (1) $$\frac{Movement\;playing\;time}{\;On\;court\;playing\;time}*100.$$
- Performance Index Rating (PIR). This index is used to assess the player rating in each match via the total performance and the global efficacy in technical actions. ACB, LEB, Euroleague, and Eurocup rate players’ performance with this index. Finally, for comparison purposes, the individual rating was normalized concerning the movement playing time of each player as follows:
  (2) $$\frac{\begin{array}{c} \left(Points+Rebounds+Assists+Steals+Blocks+Fouls\;Drawn\right)-\\ \left(Missed\;Field\;Goals+Missed\;Free\;Throws+Turnovers+Shots\;Rejected+Fouls\;Commited\right)\; \end{array}}{Movement\;playing\;time}.$$
- Internal load. From the heart rate raw data and the maximum reached in competition, different variables were calculated: (a) average heart rate (HR_AVG_, bpm); (b) maximum heart rate (HR_MAX_, bpm); (c) maximum heart rate percentages (*very low* [50%–60%] HR_MAX_; *low* [60%–70%]HR_MAX_; *moderate* [70%–80%] HR_MAX_; *high* [80%–90%] HR_MAX_; *very high* [90%–95%] HR_MAX_; *maximum* [95%–100%] HR_MAX_).
- External load. In order to compare results among playing positions, quarters and results per quarters, variables were selected related to playing time per minute: (a) Player Load, accumulated accelerometer load in the three axes of movement (PL, a.u./min); (b) total impacts >3G (ImpT, *n*/min); (c) impacts at different intensity ranges (*low*, [3–5G]Imp; *moderate*, [3–5G]Imp; *high*, [>8G]Imp); (d) steps (*n*/min); (e) jumps (*n*/min).

### 2.5. Procedures

The data were recorded during the regular league of the 2018–2019 regional-level season in the same court (five at-home and one away games). Before the beginning of the study, a familiarization with high monitoring was done by registering a training week (three sessions). The inertial devices, HR bands and video recordings were placed 40 min before the warm-up and start of the games. A synced and calibration process was performed after the registration following the manufacturer’s guidelines. For this, the inertial devices were switched on in a flat zone, being static for 30 s and without magnetic devices around [11]. Inertial devices were kept in a customized harness on the middle line between the scapulae at the C7 level, fitted tightly to the body as is typical in matches [7]. HR sensors were placed by chest bands and a video camera was set up on a tripod at the center of the court. When the registration ended, all devices were removed, and the extraction of the data was performed using the manufacturer’s software.

Finally, the registering of the technical actions of each match was performed by one observer through video analysis after the match. For this process, the observer was trained following previous methodological protocol [29]. This process was composed of four stages: preparatory, selection of observers, observers’ training and reliability processing. The within-coder reliability was realized through the Cohen’s Kappa coefficient; 108 technical actions corresponding to two quarter periods were analyzed as sample units. The Cohen’s Kappa coefficient obtained a value of 0.93, meaning the level of reliability and agreement was almost perfect (>0.80) [30].

### 2.6. Statistical Analysis

Firstly, a descriptive analysis (mean and standard deviation; M ± SD) was performed. The data distribution and the homogeneity of variance were calculated through the Kolmogorov-Smirnov and Levene tests, obtaining a non-parametric distribution [31]. Therefore, Kruskal-Wallis’ H with Mann-Whitney’s U post hoc tests were performed to analyze the influence of independent variables on workload demands and technical performance. The effect sizes were obtained by epsilon partial square ($E_{R}^{2}$) and Cohen’s d (*d*). $E_{R}^{2}$ was interpreted as follows: >0.01 *low*; >0.06 *moderate* and >0.14 *high*; and *d* was interpreted as: *d* > 0.2 *low*, *d* > 0.5 *moderate*, and *d* > 0.8 *high* [32].

Finally, a correlational analysis to identify relationships between (a) technical and internal workload, (b) technical and external workload, and (c) internal and external workload in amateur-level basketball players was performed using the Spearman correlation coefficient, interpreted as follows: *insignificant* (*r_s_* < 0.1), *low* (0.1 < r_s_ < 0.3), *moderate* (0.3 < *r_s_* < 0.5), *high* (0.5 < *r_s_* < 0.7), *very high* (0.7 < *r_s_* < 0.9), *almost perfect* (*r_s_* > 0.9) and *perfect* (*r_s_* = 1) [31]. The significance level was established at *p* < 0.05. Data analysis was performed by Statistical Package for the Social Science (SPSS Statistics, version 24, IBM Corporation, Armonk, NY, USA).

## 3. Results

### 3.1. Playing Position

Table 1 shows the analysis of internal and external workload and technical performance depending on the player’s position within the team. With respect to the effective on-court time, guards had significantly higher values than centers (*p* = 0.01; *d* = 0.49 *low*). However, centers had greater PIR than forwards (*p* < 0.02; *d* = 0.51 *moderate*). With respect to internal demands, guards (*p* < 0.05; *d* = 0.44 *low*) and centers (*p* < 0.02; *d* = 0.20 *low*) presented statistically significant differences to forwards in HR_AVG_. However, no statistical differences were found between playing positions in terms of percentages of HR_MAX_; forwards had greater duration at [70%–80%] HR_MAX_, guards at [90%–95%] HR_MAX_ and centers at [95%–100%] HR_MAX_.

In terms of external workload demands, guards presented higher PL than forwards (*p* = 0.01; *d* = 0.75 *moderate*) and centers (*p =* 0.01; *d* = 0.2 *low*). In Imp, no differences were found. In terms of intensity, guards (*p* = 0.01; *d* = 0.63 *moderate*) and forwards (*p* < 0.03; *d* = 0.62 *moderate*) suffered more low-intensity impacts [3–5G]Imp, while centers received more moderate [5–8G]Imp (Center > Guard > Forward) and high-intensity impacts [>8G]Imp (Center > Guard > Forward). Finally, guards (*p* = 0.01; *d* = 1.29 *high*) and forwards (*p* = 0.01; *d* = 1.07 *high*) performed more steps than centers, while centers performed the highest number of jumps (Centers > Guards > Forwards).

### 3.2. Accumulated Point Differences of Each Period

Table 2 presents the results of external and internal workload and technical performance related to the accumulated point differences of each period. The effective on-court time was higher in unbalanced quarters as opposed to balanced and close matches, with a low effect size. While the PIR between quarters did not attain significant difference, the highest values were seen in balanced quarters. With respect to the internal workload, statistical differences were found in HR_AVG_ and at different intensities. Close games presented greater values of maximum demand [95%–100%] HR_MAX_, balanced games of low to moderate intensity [50%–80%] HR_MAX_ and unbalanced games of high intensity [80%–95%] HR_MAX._

Finally, in terms of the external workload, no statistical differences were present between groups for all variables analyzed, except for the number of steps, which was higher for close and balanced quarters than for unbalanced quarters.

### 3.3. Final Score Difference for Each Period

The results of external and internal workload and technical performance, related to the final score difference of each period, are shown in Table 3. No differences were found between the point difference at the end of each quarter. Instead, in terms of both PIR and internal load, the highest demands were found in close and balanced quarters.

### 3.4. Period of Play

Table 4 shows the analysis of internal and external workload demands and the technical performance as a function of the period of play. Significant differences were found in the effective on-court time between the first half and second half (1° = 2° > 3° = 4°), with a moderate effect size (*d* = 0.50–0.73). However, no differences were found in PIR between quarters.

In the internal load analysis, higher values were found in Q4 with respect to Q2 in HR_AVG_ (*p <* 0.02; *d* = *0*.53 *moderate*) and [50%–60%] HR_MAX_ (*p* < 0.17; *d* = 0.34 *low*) and in [95%–100%] HR_MAX_ in Q1 and Q2 with respect to Q4. However, no differences were found in the resting heart rate percentages, with maximum values of [70%–80%] HR_MAX_ and [80%–90%] HR_MAX_ in Q3 and [90%–95%] HR_MAX_ in Q4. With respect to the external load, no differences were found in any variable. The highest values of [3–5G]Imp and steps were found in Q1, [5–8G]Imp and [>8G]Imp in Q2, and PL and jumps in Q4.

### 3.5. Correlations among Physical and Technical Variables

Finally, a correlational analysis was performed to identify relationships between (a) technical and internal workload, (b) technical and external workload, and (c) internal and external workload in amateur-level basketball players, as shown in Table 5. In terms of technical performance, the effective on-court time had moderate correlations with high-intensity internal load demands HR_AVG_ (*r_s_* = 0.49) and [95%–100%] HR_MAX_ (*r_s_* = 0.44), and external low-intensity workload demands ImpT (*r_s_* = 0.30) and [3–5G]Imp (*r_s_* = 0.41). However, the performance index rating did not have a statistically significant relationship with any variable.

On the other hand, in terms of physical performance, the high-intensity internal demands (HR_AVG_ and [95%–100%] HR_MAX_) had a high correlation with external workload demands ImpT, [3–5G]Imp, [5–8G]Imp, [>8G]Imp and Jumps (*r*_s_ = 0.17–0.40). No relationship was found between internal load and PL and steps. With respect to the external workload analysis, PL was related to ImpT, [3–5G]Imp, [5–8G]Imp, [>8G]Imp and steps (*r_s_* = 0.47–0.66), but not to jumps. Jumps were explained by high-intensity impacts ([5–8G]Imp and [>8G]Imp) (*r*_s_ = 0.40–0.42).

## 4. Discussion

An analysis of workload demands and technical performance in amateur basketball is necessary as a first approach due to limited access to technology. Furthermore, it is necessary to evaluate different contextual variables such as playing positions, periods of play, accumulated point difference throughout the quarters, acute point difference at each quarter and the relationship between technical and physical performance in senior amateur basketball players, with the aim of designing specific training sessions and individualizing workload demands. The main results indicated an influence of the three contextual variables playing position, match period and accumulated point differences through quarters on the physical and technical performance in competition, except in acute quarters.

The total workload and technical performance are related to the playing position in different ways. Guards had a faster effective on-court time, more displacements, and a higher internal and external workload at low intensity. Forwards presented similar demands to guards, but with lower values. Finally, centers obtained the best technical performance and put in more effort at maximum intensity (jumps, impacts, HR). These results are similar to others previously obtained in elite male basketball, both in the youth [7,33] and senior categories [4,34]. These studies indicated that guards covered a greater distance at high intensity [4,35], searching for free areas outside the three-point line and moving around the court from side to side [19], and also controlled the game at a technical/tactical level [36]. Forward is a playing position that presents medium values in physical and technical performance, with a prevalence of offensive tasks and three-point field-goals [36,37]. The center is the tallest player, plays near the basket and performs most of the static high-intensity impacts, contacts, and collisions with opponents [38]; because of their role they need to perform specific actions in the free throw lane [3]. Therefore, for amateur basketball players, the specific physical profile of each playing position is similar to that for elite-level basketball players. However, in terms of technical performance, the teams with higher performance are those that have more efficient centers on their roster due to their greater influence on the performance index rating. This is produced by playing closer to the basket, which allows for more rebounds, greater two-pointer efficacy, more fouls drawn and free-throw shooting [39]. In basketball, the technical actions that contribute the most to winning a game/tournament are efficient two-pointers and free throws, and defensive rebounds [36,40]. Therefore, these should be part of the strategy planning during a competition.

The point difference accumulated throughout a game’s quarters had an influence on the effective on-court time, with higher values in unbalanced games with respect to balanced and close games, but no differences were found in terms of the performance index rating. Conversely to this research, previous studies have found differences in technical performance in elite-level basketball players, with the worst performance seen in losing teams during unbalanced games [40,41]. In addition, the worst-ranking teams are those with the least efficient offense [26]. So, the different results found in this study could be due to the lower technical proficiency of amateur basketball players. As for the internal workload, differences were found in the HR_AVG_ and percentages of HR_MAX_. During an unbalanced situation, the intensity was moderate to high [60%–95%] HR_MAX_, while in balanced and close quarters the intensity was maximum [95%–100%] HR_MAX_. As for the external workload, no significant correlation with points differences was found for any variable. During close and balanced quarters, an increase in heart rate did not affect the external workload. This could be due to the maintaining of intensity and competitive stress due to a low difference in points on the scoreboard. So, the functional demands of different scenarios could be influenced by lower synchronization [34], lower defensive pressure [42] and changes in effective on-court time [40,43], where unbalanced games produced a faster rhythm. Previous research has found that the best-ranking teams in tournaments have fewer possessions, make more effective attacks, and perform positional attacks near the maximum possession time (close to 24 s) [26]. Therefore, the accumulated point difference influences amateur basketball game dynamics and conditioned-result training tasks should be developed to prepare for competition on the physical and psychological levels.

With respect to quarters, the highest effective on-court time and performance index rating were found in the first quarter. The second quarter is characterized by greater HR_AVG_ and more total impacts at high intensity. However, in the second half (Q3 and Q4), a decreasing performance was found in terms of HR, but no differences were found in terms of external workload, with a small performance increase in the last quarter. At a technical level, the best performance index rating and the highest effective on-court time were found in the first quarter, which could be due to lower defensive pressure (fewer blocks and fouls) obtained in a study during the National Basketball Association (NBA) competition [39] or a lower influence of fatigue on decision-making [44]. As for internal physical performance, a decrease in HR_AVG_ in Q4 with respect to Q1, Q2 and Q3 [19] was found, which could be due to the accumulation of blood lactate at half time [20], which increases in the second half. Although in the final periods (Q3 and Q4) of basketball games there are more stoppages and timeouts [45], these actions do not allow players to recover to optimal levels in the second half [46], especially amateur basketball players with a poorer physical performance.

However, no between-quarter differences were found in terms of the external workload. Previous studies found a better physical performance in the first quarter and a decline throughout the further game quarters, with the greatest difference being between Q1 and Q2 in youth elite basketball [7]. In this sense, Abdelkrim et al. [19] showed a decrease in the amount of high-intensity activity in the last quarter in elite under-19-year-old basketball players (*p* < 0.01; 22.41%). Other studies confirm these findings, but no data comparability was reported [2,47]. In female basketball, a similar dynamic to the present study was found. Delextrat et al. [34] failed to find differences between quarters, reporting only a small effect size between the first and third to last quarters (*d* = 0.1). These contrasting results were found in other previous studies that seem to be all on female players [11,48]. Moreover, these demands could be influenced according to the match status, with different paces and strategies [25], which translates into different activity levels according to the team’s needs. For example, in the last quarter, winning teams want to control the ball to avoid quick transitions and score easily [19]. On the other hand, losing teams want to reduce the score difference. Finally, stoppage durations have a direct influence on activity demands according to the playing level [48]. Therefore, in amateur basketball, a decrease in effective on-court time and internal load performance was found throughout the game periods, but no effect on external workload was seen. These results could be due to the lower physical prowess of nonprofessional players producing more fluctuations in performance than in professional players throughout the game.

Finally, in the analysis of the relationship between physical and technical performance, in contrast with professional-level basketball, it is identified that technical efficacy (PIR) is not related to physical performance (internal and external load). In this sense, the technical performance of amateur basketball players is not conditioned by the physical level. Thus, in these categories we can find a wide variety of player profiles (poor physical condition and good technical performance, good physical condition and poor technical performance; or average values of physical condition and technical performance). On the other hand, there is a correlation between low-intensity heart rates concerning the number of steps and player load (volume) and between high-intensity heart rate with high-intensity impacts and jumps (intensity) [5,9]. Therefore, in the amateur category, the training must be focused on the overall quality of the team (physical vs. technical aspects). At a physical level, this requires designing specific tasks oriented to a continuous workload (volume) or an intermittent workload (intensity). For volume workload development, full-court tasks should be designed whereby the player has to make coast-to-coast displacements to generate the greatest number of steps and player load with low-intensity impacts and a moderate percentage of maximum heart rate (e.g., fast break with different number of players and rules, usually with the numerical superiority of the attacking team). On the other hand, for intensity workload development, it is recommended to design half-court tasks to generate high-intensity impacts, collisions and jumps (e.g., small-sided games: 1 vs. 1, 2 vs. 2, 3 vs. 3, etc.).

While this study has provided the first workload profile of male amateur-level basketball players during official competition, and described the technical efficacy and the interaction between physical and technical performance, considering different contextual variables through inertial devices, some limitations must be acknowledged. One of the limitations concerns the sample studied. Although the target study population is larger at this level, the lack of knowledge of coaches and players and their refusal to change the work model caused a low response rate. This meant that only one team with a specific game system provided the results of the study. Future research should analyze the workload demands in amateur-level basketball, in both male and female players, with the aim of expanding scientific knowledge in this area, which will be useful in the future for new players and for coaches involved in training design and strategic planning.

## 5. Conclusions and Practical Applications

Based on the results obtained in the present study, different conclusions and practical applications can be established. In amateur-level basketball, players’ characteristics influence the workload demands during a competition. Guards present a higher volume of displacements and accelerate the game rhythm, while centers perform more static actions with collisions and have better technical performance. It is necessary to adapt the training sessions workload according to the requirements of each specific position to achieve the best performance.

A decrease in physical performance was found between the two halves (1st half: Q1 and Q2; 2nd half: Q3 and Q4) of the game, identified by lower effective on-court time and intensity (HR_AVG_ and %HR_MAX_), while no between-quarter differences were found in terms of external workload. To avoid the effects of fatigue, inter-period recovery strategies should be planned by medical staff and the total playing time should be distributed among the players throughout the match periods, especially for amateur basketball players since they have lower physical endurance with respect to elite-level basketball players.

With respect to the match status, an unbalanced accumulated difference of points in the quarters produced lower technical performance and lower internal and external workload demands. However, no differences in physical and technical performance were found in terms of the acute points difference in each quarter. Due to the influence of the accumulated point differences of each period throughout the match, it is necessary to design game-based tasks with modified rules based on the match status (winning, losing or drawing) and points difference (close, balanced and unbalanced) so that players can better face these scenarios in competitions.

Finally, no relationship was found between technical and physical performance. In terms of physical performance variables, a higher effective on-court time had a direct correlation with internal and external workload demands. Therefore, the monitoring and analysis of the physical and technical performance in amateur-level senior basketball will allow for the design of training loads, planning strategies, injury prevention programs and recovery protocols between workouts and matches tailored to this specific level. In this sense, due to not finding a relationship between the physical and technical performance in amateur basketball, the effective on-court time during the competition will depend on the specific characteristics of team players (technical vs. physical level). In this sense, games and training should follow one of two strategies: (a) play fast with great physical demands to fatigue the opponent and get more exchange of possessions during the game or (b) focus training on improving all technical elements to score the greatest number of points in a static attack.

## Figures and Tables

**Table 1:** Descriptive and inferential analysis of the analyzed variables as a function of playing position.

Variables	Guards (*n* = 60)	Forwards (*n* = 52)	Centers (*n* = 67)	*p*	*χ*²	$E_{R}^{2}$	*Magnitude*
	M ± SD 95%CI (L–U)	M ± SD 95%CI (L–U)	M ± SD 95%CI (L–U)				
Effective on-court time (%)	45.03 ± 11.29 ^‡^ (42.34–47.73)	42.86 ± 11.81 (39.78–45.94)	39.69 ± 10.49 * (37.29–42.09)	0.01	9.88	0.05	*low*
PIR (a.u./min)	0.14 ± 0.64 (−0.02–0.30)	0.04 ± 0.81 ^‡‡^ (−0.18–0.26)	0.45 ± 0.81 ^††^ (0.25–0.64)	0.02	8.22	0.04	*low*
HR_AVG_ (bpm)	175.76 ± 10.61 ^†^ (173.23–178.29)	169.52 ± 17.09 * ^‡^ (164.85–174.18)	173.25 ± 20.22 ^†^ (168.39–178.10)	0.02	8.30	0.04	*low*
[50%–60%] HR_MAX_	0.17 ± 1.28 (−0.13–0.48)	2.06 ± 13.60 (−1.65–5.77)	1.40 ± 7.99 (−0.52–3.32)	0.13	3.92	0.00	
[60%–70%] HR_MAX_	1.09 ± 4.14 (0.11–2.08)	2.15 ± 4.59 (0.90–3.41)	3.11 ± 11.62 (0.32–5.91)	0.09	4.78	0.02	*low*
[70%–80%] HR_MAX_	7.09 ± 13.05 (3.98–10.20)	8.07 ± 8.22 (5.83–10.31)	5.98 ± 6.57 (4.40–7.56)	0.17	3.66	0.00	
[80%–90%] HR_MAX_	16.60 ± 12.79 (13.55–19.65)	15.53 ± 11.85 (12.29–18.76)	13.95 ± 10.88 (11.34–16.57)	0.50	1.42	0.00	
[90%–95%] HR_MAX_	16.53 ± 9.71 (14.22–18.85)	16.61 ± 9.77 (13.94–19.28)	13.98 ± 11.53 (11.21–16.75)	0.08	4.96	0.01	*low*
[95%–100%] HR_MAX_	58.52 ± 26.40 (52.22–64.81)	53.64 ± 25.38 (46.71–60.78)	59.58 ± 28.78 (52.66–66.49)	0.25	2.75	0.00	
PL (a.u./min)	3.44 ± 0.35 ^†† ‡^ (3.36–3.53)	3.19 ± 0.32 ** (3.11–3.28)	3.24 ± 0.29 * (3.17–3.31)	0.01	17.11	0.09	*moderate*
Imp (*n*/min)	90.86 ± 31.25 (83.36–98.37)	82.14 ± 17.49 (77.50–86.78)	88.35 ± 12.69 (85.39–91.31)	0.06	5.50		
[3–5G]Imp (*n*/min)	67.93 ± 18.66 ^‡‡^ (63.45–72.41)	65.60 ± 13.04 ^‡‡^ (62.14–69.06)	59.10 ± 6.63 ** ^††^ (57.55–60.64)	0.01	12.74	0.06	*moderate*
[5–8G]Imp (*n*/min)	20.11 ± 12.02 ^†† ‡‡^ (17.22–23.00)	15.02 ± 6.64 ** ^‡‡‡^ (13.26–16.78)	25.45 ± 8.09 ** ^†††^ (23.56–27.34)	0.01	38.03	0.19	*high*
[>8G]Imp (*n*/min)	2.84 ± 2.38 ^†† ‡^ (2.27–3.41)	1.53 ± 1.58 ** ^‡‡‡^ (1.11–1.95)	3.80 ± 3.14 * ^†††^ (3.07–4.53)	0.01	39.16	0.20	*high*
Steps (*n*/min)	180.47 ± 11.51 ^‡‡‡^ (177.70–183.23)	179.69 ± 15.11 ^‡‡‡^ (175.68–183.70)	164.27 ± 13.54 *** ^†††^ (161.11–167.42)	0.01	55.88	0.28	*high*
Jumps (*n*/min)	0.96 ± 0.43 ^†† ‡‡‡^ (0.86–1.07)	0.65 ± 0.53 ** ^‡‡‡^ (0.51–0.79)	1.77 ± 0.88 *** ^†††^ (1.56–1.97)	0.01	65.86	0.33	*high*

M: Mean; SD: Standard deviation; CI: Confidence interval (U: Upper–L: Lower); *p*: significance; *χ*²: Chi-square; $E_{R}^{2}$: partial epsilon square. * Statistical differences with guards (*p* < 0.05; effect size: * low, ** moderate, *** high). † Statistical differences with forwards (*p* < 0.05; effect size: † low, †† moderate, ††† high). ‡ Statistical differences with centers (*p* < 0.05; effect size: ‡ low, ‡‡ moderate, ‡‡‡ high).

**Table 2:** Descriptive and inferential analysis of the analyzed variables as a function of the accumulated point difference at the end of the quarters.

Variables	Close (0–15) (*n* = 77)	Balanced (15–30) (*n* = 77)	Unbalanced (>30) (*n* = 25)	*p*	*χ*²	$E_{R}^{2}$	*Magnitude*
	M ± SD 95%CI (L–U)	M ± SD 95%CI (L–U)	M ± SD 95%CI (L–U)				
Effective on-court time (%)	40.83 ± 7.07 ^‡^ (38.14–43.52)	40.01 ± 14.76 ^‡^ (36.88–43.13)	44.48 ± 9.32 * ^†^ (42.53–46.43)	0.04	6.26	0.05	*low*
PIR (a.u./min)	0.30 ± 0.74 (0.14–0.45)	2.15 ± 17.44 (−1.89–6.19)	0.24 ± 0.92 (−0.11–0.59)	0.82	0.41	0.00	
HR_AVG_ (bpm)	175.55 ± 9.99 ^†^ (173.35–177.74)	169.03 ± 23.61 * ^‡^ (164.03–174.04)	174.36 ± 9.08 ^†^ (170.61–178.11)	0.05	5.13	0.04	*low*
[50%–60%] HR_MAX_	0.14 ± 1.15 ^†^ (−0.12–0.39)	2.93 ± 15.52 * ^‡^ (−0.3–6.21)	1.61 ± 5.32 ^†^ (−0.59–3.80)	<0.01	10.56	0.10	*moderate*
[60%–70%] HR_MAX_	1.44 ± 5.38 ^† ‡‡^ (0.26–2.62)	3.45 ± 14.19 * ^‡^ (0.44–6.45)	3.88 ± 6.95 ** ^†^ (1.01–6.75)	<0.01	17.33	0.16	*high*
[70%–80%] HR_MAX_	5.32 ± 5.82 ^†† ‡^ (4.04–6.60)	8.17 ± 12.77 ** ^‡^ (5.47–10.88)	7.56 ± 7.10 * ^†^ (4.63–10.49)	0.05	5.03	0.04	*low*
[80%–90%] HR_MAX_	12.60 ± 8.05 ^† ‡‡‡^ (10.83–14.36)	15.42 ± 11.70 * ^‡‡‡^ (12.94–17.90)	22.91 ± 18.58 *** ^†††^ (15.24–30.58)	<0.01	9.77	0.09	*moderate*
[90%–95%] HR_MAX_	13.67 ± 8.27 ^† ‡‡^ (11.85–15.49)	16.19 ± 12.08 * ^‡^ (13.63–18.75)	18.92 ± 10.28 ** ^†^ (14.68–23.17)	0.02	7.81	0.07	*moderate*
[95%–100%] HR_MAX_	66.84 ± 19.65 ^†† ‡‡‡^ (62.52–71.16)	51.08 ± 29.56 ** ^‡^ (44.81–57.34)	45.11 ± 31.92 *** ^†^ (31.93–58.29)	<0.01	14.21	0.14	*high*
PL (a.u./min)	3.34 ± 0.31 (3.27–3.40)	3.31 ± 0.38 (3.23–3.40)	3.19 ± 0.40 (3.04–3.34)	0.28	2.54	0.00	
Imp (*n*/min)	87.46 ± 21.17 (83.03–91.90)	89.49 ± 22.86 (84.43–94.54)	82.80 ± 23.90 (73.70–91.89)	0.38	1.94	0.00	
[3–5G]Imp (*n*/min)	64.58 ± 14.30 (61.59–67.58)	65.79 ± 15.18 (62.43–69.15)	59.27 ± 13.22 (54.24–64.30)	0.23	2.99	0.00	
[5–8G]Imp (*n*/min)	20.41 ± 9.27 (18.47–22.35)	20.77 ± 10.88 (18.36–23.17)	20.08 ± 11.66 (15.65–24.52)	0.67	0.81	0.00	
[>8G]Imp (*n*/min)	2.46 ± 1.94 (2.06–2.87)	2.93 ± 2.83 (2.31–3.56)	3.48 ± 3.82 (2.03–4.94)	0.40	1.81	0.00	
Steps (*n*/min)	176.63 ± 16.51 ^‡‡^ (173.18–180.09)	175.30 ± 17.83 ^‡‡^ (171.35–179.24)	167.59 ± 12.29 ** ^††^ (162.92–172.27)	0.05	5.47	0.04	*low*
Jumps (*n*/min)	1.15 ± 0.77 (0.99–1.31)	1.13 ± 0.83 (0.95–1.32)	1.29 ± 0.86 (0.96–1.61)	0.85	0.34	0.00	

M: Mean; SD: Standard deviation; CI: Confidence interval (U: Upper–L: Lower); *p*: significance; *χ*²: Chi-square; $E_{R}^{2}$: partial epsilon square. * Statistical differences with close quarters (*p* < 0.05; effect size: * low, ** moderate, *** high). † Statistical differences with balanced quarters (*p* < 0.05; effect size: † low, †† moderate, ††† high). ‡ Statistical differences with unbalanced quarters (*p* < 0.05; effect size: ‡ low, ‡‡ moderate, ‡‡‡ high).

**Table 3:** Descriptive and inferential analysis of the analyzed variables as a function of the point difference at the end of the quarters.

Variables	Close (0–7) (*n* = 36)	Balanced (7–15) (*n* = 57)	Unbalanced (>15) (*n* = 76)	*p*	*χ*²	$E_{R}^{2}$	*Magnitude*
	M ± SD 95%CI (L–U)	M ± SD 95%CI (L–U)	M ± SD 95%CI (L–U)				
Effective on-court time (%)	42.72 ± 7.44 (41.23–44.21)	44.57 ± 7.44 (42.58–46.56)	47.96 ± 6.21 (43.79–52.13)	0.06	5.89	0.00	
PIR (a.u./min)	0.33 ± 0.64 (0.20–0.45)	0.16 ± 0.53 (0.01–0.31)	−0.04 ± 0.58 (−0.42–0.35)	0.13	4.16	0.00	
HR_AVG_ (bpm)	175.82 ± 8.65 (174.02–177.63)	177.06 ± 8.45 (174.75–179.36)	179.09 ± 8.15 (173.61–184.57)	0.47	1.53	0.00	
[50%–60%] HR_MAX_	0.44 ± 2.84 (−0.15–1.03)	0.04 ± 0.23 (−0.02–0.10)	0.00 ± 0.00 (0.00–0.00)	0.82	0.39	0.00	
[60%–70%] HR_MAX_	1.44 ± 4.02 (0.60–2.28)	0.38 ± 0.94 (0.13–0.64)	1.08 ± 1.90 (−0.19–2.36)	0.24	2.85	0.00	
[70%–80%] HR_MAX_	6.06 ± 7.89 (4.42–7.71)	5.67 ± 6.01 (4.03–7.31)	8.94 ± 5.06 (5.54–12.34)	0.08	4.98	0.00	
[80%–90%] HR_MAX_	15.95 ± 12.53 (13.34–18.56)	15.47 ± 10.92 (12.49–18.46)	20.59 ± 13.03 (11.84–29.34)	0.38	1.95	0.00	
[90%–95%] HR_MAX_	15.76 ± 9.31 (13.82–17.70)	16.51 ± 11.06 (13.49–19.53)	18.89 ± 11.38 (11.24–26.53)	0.74	0.60	0.00	
[95%–100%] HR_MAX_	60.34 ± 24.69 (55.20–65.49)	61.92 ± 23.45 (55.52–68.32)	50.50 ± 25.35 (33.48–67.53)	0.31	2.36	0.00	
PL (a.u./min)	3.36 ± 0.32 (3.29–3.42)	3.30 ± 0.34 (3.21–3.39)	3.15 ± 0.26 (2.97–3.33)	0.10	4.39	0.00	
Imp (*n*/min)	89.18 ± 21.58 (84.85–93.51)	87.98 ± 23.77 (81.62–94.35)	88.31 ± 22.08 (73.47–103.14)	0.95	0.11	0.00	
[3–5G]Imp (*n*/min)	64.61 ± 14.05 (61.80–67.43)	63.47 ± 14.66 (59.55–67.40)	63.80 ± 11.81 (55.86–71.73)	0.92	0.17	0.00	
[5–8G]Imp (*n*/min)	21.52 ± 9.96 (19.52–23.52)	21.50 ± 10.35 (18.73–24.27)	21.37 ± 11.73 (13.49–29.25)	0.99	0.03	0.00	
[>8G]Imp (*n*/min)	3.05 ± 2.70 (2.50–3.59)	3.04 ± 2.82 (2.28–3.79)	3.14 ± 3.09 (1.06–5.22)	0.97	0.06	0.00	
Steps (*n*/min)	174.24 ± 14.25 (171.38–177.09)	176.02 ± 15.04 (172.00–180.05)	172.42 ± 11.41 (164.76–180.09)	0.61	1.01	0.00	
Jumps (*n*/min)	1.25 ± 0.78 (1.10–1.41)	1.03 ± 0.66 (0.85–1.20)	1.07 ± 0.84 (0.50–1.63)	0.13	4.05	0.00	

M: Mean; SD: Standard deviation; CI: Confidence interval (U: Upper–L: Lower); *p*: significance; *χ*²: Chi-square; $E_{R}^{2}$: partial epsilon square.

**Table 4:** Descriptive and inferential analysis of the analyzed variables in function of the match period.

Variables	Period 1 (*n* = 45)	Period 2 (*n* = 46)	Period 3 (*n* = 43)	Period 4 (*n* = 45)	*p*	*χ*²	$E_{R}^{2}$	*magnitude*
	M ± SD 95%IC (L–U)	M ± SD 95%IC (L–U)	M ± SD 95%IC (L–U)	M ± SD 95%IC (L–U)				
Effective on-court time (%)	46.22 ± 9.58 ^‡‡^ ⁑⁑ (43.53–48.92)	45.44 ± 10.01 ^‡‡^ ⁑⁑ (42.63–48.26)	40.45 ± 10.15 ** ^††^ (37.53–43.36)	37.79 ± 13.11 ** ^††^ (34.21–41.37)	0.01	13.33	0.07	*moderate*
PIR (a.u./min)	0.40 ± 0.76 (0.19–0.62)	0.16 ± 0.66 (−0.03–0.35)	0.13 ± 0.63 (−0.06–0.31)	0.19 ± 1.0 (−0.12–0.50)	0.32	3.53	0.02	*low*
HR_AVG_ (bpm)	175.81 ± 10.68 (172.71–178.91)	176.51 ± 12.44⁑⁑ (172.94–180.08)	174.40 ± 11.32 (171.0–177.80)	166.18 ± 24.65 ^††^ (159.24–173.11)	0.14	10.60	0.05	*low*
[50%–60%] HR_MAX_	0.08 ± 0.37( −0.03–0.19)	0.00 ± 0.00 ⁑ (0.00-0.00)	0.23 ± 1.54 (−0.23–0.69)	4.03 ± 16.55 ^†^ (−0.62–8.68)	0.16	10.33	0.05	*low*
[60%–70%] HR_MAX_	2.32 ± 6.68 (0.37–4.26)	2.30 ± 2.30 (−1.17–5.77)	1.50 ± 4.96 (0–2.99)	2.29 ± 5.33 (0.79–3.79)	0.27	3.95	0.02	*low*
[70%–80%] HR_MAX_	6.65 ± 7.18 (4.56–8.73)	6.29 ± 6.29 (4.37–8.21)	8.06 ± 14.11 (3.82–12.30)	6.95 ± 9.99 (4.14–9.76)	0.79	1.06	0.00	
[80%–90%] HR_MAX_	13.91 ± 9.64 (11.11–16.71)	15.06 ± 15.06 (11.71–18.41)	16.44 ± 13.24 (12.46–20.42)	16.03 ± 12.83 (12.42–19.63)	0.87	0.74	0.00	
[90%–95%] HR_MAX_	14.61 ± 9.03 (11.98–17.23)	15.15 ± 15.15 (12.07–18.24)	16.13 ± 10.75 (12.90–19.36)	16.65 ± 11.24 (13.49–19.81)	0.77	1.11	0.00	
[95%–100%] HR_MAX_	62.34 ± 23.89 ⁑ (55.40–69.28)	61.20 ± 21.20 ⁑ (53.87–68.53)	57.65 ± 27.52 (49.38–65.91)	49.38 ± 29.39 * † (41.11–57.64)	0.08	6.53	0.03	*low*
PL (a.u./min)	3.26 ± 0.36 (3.16–3.36)	3.28 ± 3.28 (3.18–3.37)	3.29 ± 0.36 (3.19–3.40)	3.35 ± 0.29 (3.27–3.44)	0.58	1.98	0.00	
Imp (*n*/min)	87.58 ± 23.57 (80.95–94.21)	89.03 ± 89.03 (82.70–95.35)	86.16 ± 22.47 (79.64–92.68)	86.95 ± 21.01 (80.98–92.92)	0.82	0.94	0.00	
[3–5G]Imp (*n*/min)	64.50 ± 15.0 (60.28–68.72)	64.23 ± 64.23 (60.18 – 68.28)	62.91 ± 13.49 (59.0–66.83)	64.39 ± 13.92 (60.43–68.35)	0.92	0.51	0.00	
[5–8G]Imp (*n*/min)	20.46 ± 10.99 (17.37–23.55)	21.86 ± 21.86 (19.03–24.69)	20.45 ± 9.97 (17.56–23.35)	19.67 ± 9.91 (16.86–22.49)	0.72	1.33	0.00	
[>8G]Imp (*n*/min)	2.62 ± 2.57 (1.90–3.35)	2.94 ± 2.94 (2.11–3.77)	2.82 ± 2.67 (2.04–3.59)	2.89 ± 2.53 (2.17–3.60)	0.66	1.57	0.00	
Steps (*n*/min)	175.79 ± 15.78 (171.35–180.23)	172.22 ± 172.22 (168.39–176.05)	174.05 ± 16.79 (169.17–178.92)	175.11 ± 15.42 (170.73–179.50)	0.64	1.71	0.00	
Jumps (*n*/min)	1.17 ± 0.82 (0.94–1.40)	1.18 ± 1.18 (0.98–1.38)	1.08 ± 0.80 (0.85–1.31)	1.25 ± 0.89 (0.99–1.50)	0.82	0.93	0.00	

M: Mean; SD: Standard deviation; CI: Confidence interval (U: Upper–L: Lower); *p*: significance; *χ*²: Chi-square; $E_{R}^{2}$: partial epsilon square. *Statistical differences with first quarter (*p* < 0.05; effect size: * low, ** medium, *** high); † Statistical differences with second quarter (*p* < 0.05; effect size: † low, †† medium, ††† high); ‡ Statistical differences with third quarter (*p* < 0.05; effect size: ‡ low, ‡‡ medium, ‡‡‡ high) ⁑ Statistical differences with fourth quarter (*p* < 0.05; effect size: ⁑ low, ⁑⁑ medium, ⁑⁑⁑ high).

**Table 5:** Correlational analysis of the physical and technical variables registered in the present study.

Variables	Effective On-Court Time (%)	HR_AVG_ (bpm)	[50%–60%] HR_MAX_	[60%–70%] HR_MAX_	[70%–80%] HR_MAX_	[80%–90%] HR_MAX_	[90%–95%] HR_MAX_	[95%–100%] HR_MAX_	PL (a.u./min)	Imp (*n*/min)	[3–5G] Imp (*n*/min)	[5–8G] Imp (*n*/min)	[>8G] Imp (*n*/min)	Steps (*n*/min)	Jumps (*n*/min)
PIR (a.u/min)	−0.02	0.04	0.01	−0.02	−0.08	−0.07	−0.07	0.06	0.07	0.09	0.05	0.09	0.07	−0.06	0.12
Effective on-court time (%)		0.49 **	−0.27 **	−0.22 **	−0.31 **	−0.24 **	−0.09	0.44 **	−0.12	0.30 **	0.41 **	0.12	0.02	−0.01	−0.09
HR_AVG_ (bpm)			−0.33 **	−0.45 **	−0.61 **	−0.52 **	−0.40 **	0.79 **	0.01	0.40 **	0.33 *	0.38 **	0.22 **	0.06	0.17 *
[50%–60%] HR_MAX_				0.28 **	0.12	0.03	0.01	−0.27 **	0.01	−0.10	−0.22 **	−0.06	−0.09	0.03	−0.04
[60%–70%] HR_MAX_					0.56 **	0.34 **	0.24 **	−0.48 **	−0.06	−0.22 **	−0.21 **	−0.15 *	−0.09	−0.20 **	−0.06
[70%–80%] HR_MAX_						0.61 **	0.34 **	−0.68 **	−0.11	−0.23 **	−0.22 **	−0.19 **	−0.05	−0.23 **	−0.05
[80%–90%] HR_MAX_							0.66 **	−0.73 **	0.04	−0.15 *	−0.20 **	−0.07	0.06	−0.11	−0.06
[90%–95%] HR_MAX_								−0.63 **	0.01	−0.21 **	−0.24 **	−0.14	0.02	0.01	−0.07
[95%–100%] HR_MAX_ (bpm)									0.01	0.23 **	0.26 **	0.15 *	0.01	0.13	0.06
PL (a.u./min)										0.66 **	0.47 **	0.58 **	0.60 **	0.54 **	0.06
Imp (*n*/min)											0.83 **	0.83 **	0.67 **	0.18 **	0.22 **
[3–5G]Imp (*n*/min)												0.44 **	0.29 **	0.24 **	−0.05
[5–8G]Imp (*n*/min)													0.82 **	−0.02	0.42 **
[>8G]Imp (*n*/min)														−0.05	0.40 **
Steps (*n*/min)															−0.34 **
Interpretation of correlational values (*r_s_*) through color scale															
*Insignificant* (*r_s_* < 0.1)			*Low* (0.1 < *r_s_* < 0.3)		*Moderate* (0.3 < *r_s_* < 0.5)		*High* (0.5 < *r_s_* < 0.7)		*Very high* (0.7 < *r_s_* < 0.9)		*Almost perfect* (*r_s_* > 0.9)		*Perfect* (*r_s_* = 1)		
						

* *p* < 0.05; ** *p* < 0.01.
